# Pharmacological effects of higenamine based on signalling pathways and mechanism of action

**DOI:** 10.3389/fphar.2022.981048

**Published:** 2022-09-15

**Authors:** De-ta Chen, Wu Rao, Xue Shen, Lin Chen, Zi-jian Wan, Xiao-ping Sheng, Tian-you Fan

**Affiliations:** ^1^ Shanghai Municipal Hospital of Traditional Chinese Medicine, Shanghai University of Traditional Chinese Medicine, Shanghai, China; ^2^ Shuguang Hospital Affiliated to Shanghai University of Traditional Chinese Medicine, Shanghai, China

**Keywords:** higenamine, mechanism of action, pharmacological effect, signalling pathway, TCM

## Abstract

Higenamine (HG) is a chemical compound found in various plants, such as aconite. Recent pharmacological studies have demonstrated its effectiveness in the management of many diseases. Several mechanisms of action of HG have been proposed; however, they have not yet been classified. This review summarises the signalling pathways and pharmacological targets of HG, focusing on its potential as a naturally extracted drug. Articles related to the pharmacological effects, signalling pathways and pharmacological targets of HG were selected by searching the keyword “Higenamine” in the PubMed, Web of Science and Google Scholar databases without limiting the search by publication years. HG possesses anti-oxidant, anti-apoptotic, anti-inflammatory, electrophysiology regulatory, anti-fibrotic and lipid-lowering activities. It is a structural analogue of catecholamines and possesses characteristics similar to those of adrenergic receptor ligands. It can modulate multiple targets, including anti-inflammation- and anti-apoptosis-related targets and some transcription factors, which directly or indirectly influence the disease course. Other naturally occurring compounds, such as cucurbitacin B (Cu B) and 6-gingerol (6-GR), can be combined with HG to enhance its anti-apoptotic activity. Although significant research progress has been made, follow-up pharmacological studies are required to determine the exact mechanism of action, new signalling pathways and targets of HG and the effects of using it in combination with other drugs.

## Introduction

Fuzi, also known as Radix Aconiti lateralis praeparata, is widely used in traditional Chinese medicine to treat various disorders. The Pharmacopoeia of the People’s Republic of China states that Fuzi can restore health after syncope, reinforce fire and Yang and dispel wind and dampness. In addition, it can be used in cases in which Yang prostration has caused a faint pulse. Recent studies have shown that Fuzi can boost the immune system; has anti-shock, anti-inflammatory and anti-thrombotic properties and can be used as a cardiotonic (Zhou et al., 2015). Fuzi is composed of various alkaloids, such as diester diterpenoid alkaloids, aconitine, hypoaconitine and higenamine (HG, CAS 5843-65-2). It also contains lipids, such as fatty acids, phosphatidate calcium and steroids (Zhao et al., 2012). To prevent side effects owing to its toxic metabolites, the dosage of Fuzi should be carefully adjusted. HG is listed as an ingredient in over-the-counter weight loss and sports supplements in countries such as the United States of America; however, it is commonly used as a traditional Chinese medicine formulation in China (Cohen et al., 2019; Chang et al., 2021). HG is a benzylisoquinoline alkaloid with various pharmacological effects, including vasodilation, anti-platelet activity and calcium channel-blocking activity (Chang et al., 1994). Recent studies have shown that Fuzi acts as a β-2-adrenergic receptor agonist (Zhang N. et al., 2019), a vasodilator and smooth muscle relaxant (Bai et al., 2008) and an ion channel regulator (Wang X. et al., 2019); helps in fat and blood glucose utilisation (Kato et al., 2015) and possesses anti-inflammatory (Yang S. et al., 2020), anti-apoptotic (Yang X. et al., 2020), anti-oxidant (Wen et al., 2020a), anti-fibrotic (Deng et al., 2020), neuroregulatory (Chen S. et al., 2019) and anti-tumour activities (Jin et al., 2018). This review describes the mechanisms of action and biological activities of HG.

## Basic Properties

### Structure

**FIGURE 1:**
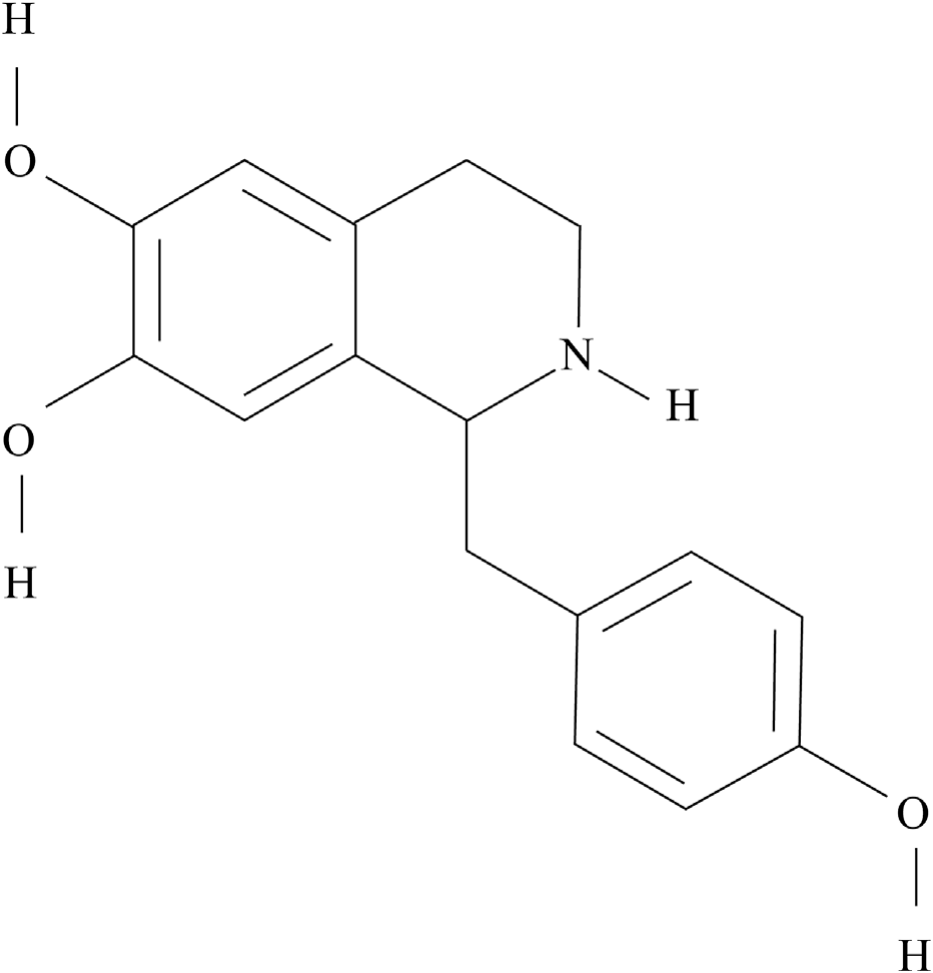
Chemical structure of HG.

HG (1-[(4-hydroxyphenyl)methyl]-1,2,3,4-tetrahydroisoquinoline-6,7-diol) is an active component of the racemic mixture isolated from Fuzi (Kosuge and Yokota, 1976). Its molecular formula and weight are C_16_H_17_NO_3_ and 271.31 g/mol, respectively. The chemical structure of HG is shown in Figure 1.

### Tolerance

The oral utilisation rate of HG is as low as 3–22% (Lo and Chen, 1996), which results in poor efficacy. Bloomer et al. (2015) and Rasic et al. (2021) observed the respiratory rate, heart rate, blood pressure and other vital signs of 48 male and 12 female volunteers after oral administration of HG and found that there were no significant changes in the indices. However, it was discovered that intravenous administration of HG increased the heart rate and left ventricular ejection fraction, indicating that this administration route may be practical for further clinical studies (Bao et al., 1982; Liu et al., 1983). Concerning the safe dosage of HG, a Chinese study on tolerance revealed that continuous intravenous infusion of HG at a rate of 4 μg/kg·min resulted in a slight increase in systolic blood pressure and a slight decrease in diastolic blood pressure but had minimal effects on the nervous system and digestive tract. These effects disappeared quickly after the cessation of drug administration (Du et al., 2007). However, further research is required to validate this phenomenon because clinical tolerance tests involving HG administration at higher doses are lacking.

### Metabolism

The half-lives of HG in rat, rabbit and human blood are approximately 8 min (Feng et al., 2012), 22 min (Lo and Chen, 1996) and 18–27 min, respectively (Wang et al., 2020; Chang et al., 2021). In a study, the average human clearance of HG was 249 L/H, renal clearance was 22.9 L/H, non-renal clearance was 226.1 L/H and the average recovery rate in urine within 8 h was 9.3%, suggesting that HG is not metabolised by the kidney but by the liver (Feng et al., 2012). A study on rats reported that 22% of HG penetrated from the blood vessels to the muscle tissue and was distributed to the interstitial fluid after an intravenous infusion of 10 mg/kg HG, which was quickly eliminated in approximately 90 min. The half-life of HG in the muscle tissues was approximately 19 min (Chang et al., 2021). However, the metabolism of HG in human muscle tissues has not been investigated. Only a few studies have described the pharmacokinetic and pharmacodynamic properties and clinical dosage of HG. Owing to the lack of clinical data, the safety, metabolic pathways and side effects of HG remain unknown. Given that HG has a fast metabolic rate and a short half-life, improving its therapeutic effects and increasing its utilisation are important directions for future research.

## Anti-apoptotic activity of higenamine

The Bcl-2 and Bax genes play an important role in determining cell survival or death (Misao et al., 1996). The release of cytochrome C from the mitochondria into the cytoplasm is a key initial step in apoptosis mediated by reactive oxygen species (ROS). HG can block the release of cytochrome C, inhibit the activation of caspase-3, reduce the expression of Bax and increase the expression of Bcl-2 protein in rats with ischaemia–reperfusion injury (Lee et al., 2006). Therefore, HG may inhibit apoptosis to some extent. The abovementioned effects can be reversed by heme oxygenase-1 (HO-1) inhibitors.

HO-1 regulates apoptosis and tissue damage (Medina et al., 2020). It is induced by various stimuli, including oxidative stress, heat shock, ultraviolet rays, ischaemia–reperfusion, heavy metals, bacterial lipopolysaccharide (LPS), cytokines, nitric oxide (NO) and heme (Shibahara, 1988). HG can upregulate HO-1 and is involved in the PI3K/AKT signalling pathway and NRF-2 translocation (Ha et al., 2012). Under normal cell conditions, NRF-2 is found in the cytoplasm and binds to the actin-binding protein KEAP-1. When electrophilic antioxidants stimulate NRF-2, it dissociates and translocates from KEAP-1 to the nucleus, where it binds to the HO-1 promoter region (Medina et al., 2020). The release of HO-1 has certain anti-inflammatory and protective effects on cells (Sakamoto et al., 2005; Giris et al., 2006; Umeda et al., 2009). High-mobility group box 1 (HMGB1) is a DNA-binding nuclear protein, which is released when stimulated by cytokines or apoptosis and is closely associated with apoptosis and inflammation (Sims et al., 2010). HO-1 regulates the expression of HMGB1, and increased HO-1 levels inhibit HMGB1 expression (Ha et al., 2011). Liu et al. (2015) found that HG increased the activity of NRF-2 and HO-1, decreased the levels of HMGB1, inhibited the activity of myeloperoxidase (MPO) and inhibited the inflammatory response to intestinal ischaemia–reperfusion injury in mice *in vivo*. However, these effects were eliminated after treatment with NRF-2 siRNA and the HO-1 inhibitor ZnPPIX. These findings suggested that the NRF-2/HO-1/HMGB1 signalling pathway is involved in the anti-apoptotic activity of HG, which is consistent with the findings of Ha et al. (2012), indicating that HG promotes HO-1 secretion and inhibits HMGB1 expression via the PI3K/AKT/NRF-2 signalling pathway to improve cerebral ischaemia–reperfusion injury. HG not only inhibits apoptosis in ischaemia–reperfusion injury but also alleviates collagen-induced arthritis by promoting HO-1 release and regulating the PI3K/AKT/NRF-2 signalling pathway (Duan et al., 2016).

The proliferation, differentiation and apoptosis of normal cells are tightly regulated by the PI3K/AKT signalling pathway. HG has been shown to increase the expression of p-PI3K and p-AKT in various tissues (Duan et al., 2016; Wu et al., 2016; An et al., 2017; Zhu J. X. et al., 2021). An et al. (2017) found that HG promotes the proliferation of gastric smooth muscle cells (SMCs) via the β2-AR/PI3K/AKT signalling pathway and inhibits apoptosis and the expression of downstream apoptosis-related targets (p-21, p-GSK3β and p-BAD). Wu et al. (2016) found that HG reduced myocardial ischemia/reperfusion (I/R) injury and cardiomyocyte apoptosis induced by hydrogen peroxide in mice by interacting with the PI3K/AKT signalling pathway. In addition, HG can activate beta-adrenergic receptor (β-AR) signal transduction (Park et al., 1984), which is involved in the PI3K/AKT signalling pathway (Wu et al., 2016; An et al., 2017). β-ARs are involved in cell apoptosis. β1-AR activation promotes apoptosis, whereas β2-AR activation inhibits cardiomyocyte apoptosis (Communal et al., 1999). HG activates β2-AR, leading to bronchodilatation, in the management of asthma (Bai et al., 2008). Wu et al. (2016) reported that HG can regulate cardiomyocyte apoptosis via the β2-AR/PI3K/AKT signalling pathway. In addition to β2-AR, ROS are involved in the PI3K/AKT signalling pathway. Zhu X. et al. (2021) reported that addition of HG downregulated ROS activity in nucleus pulposus cells stimulated with IL-1β. However, LY294002, an inhibitor of the PI3K/AKT signalling pathway, reduced the inhibitory effects of HG on apoptosis and caspase-3 activity induced by IL-1β, whereas NAC, an inhibitor of ROS, partially eliminated these effects, suggesting that HG inhibits apoptosis partly through the ROS/PI3K/AKT signalling pathway.

Furthermore, HG possesses certain anti-tumour properties. Geng and Ou (2021) reported that HG increases the expression of caspase-3, caspase-9 and Bax in tumour cells; promotes cell cycle arrest in the G2max M phase; reduces the number of cells in the S phase and promotes the apoptosis of glioma cells. In addition, HG can bind to LSD1 and downregulate H3K4 methylation, thus blocking the expression of HOX-related genes and p53 and eventually inhibiting cell apoptosis (Fang et al., 2021). As mentioned above, HG can inhibit apoptosis in some normal tissues or cells by inhibiting the expression of caspase-3 and Bax. However, whether HG plays a dual role in the caspase-3 or apoptosis-related signalling pathways remains unclear. To the best of our knowledge, no related experimental studies have been reported yet, and the relationship between HG and apoptosis warrants further investigation.

## Antioxidant activity of higenamine

The use of ROS scavengers enhances the anti-apoptotic effects of HG, which may be attributed to the strong antioxidant activity of HG (Xie et al., 2018; Romeo et al., 2020). HG inhibits the production of ROS and malondialdehyde (MDA), increases the activity of superoxide dismutase (SOD) and glutathione peroxidase (GPX) and protects cells from oxidative stress and apoptosis via the PI3K/AKT/NRF2/HO-1 signalling pathway (Zhang Y. et al., 2019). Yang S. et al. (2020) investigated the role of HG in Alzheimer’s disease and found that HG exerts antioxidant effects and can regulate the AKT/GSK3 β signalling pathway. HG increases the phosphorylation of AKT and the expression of Ser9p/GSK-3β by promoting the phosphorylation of Ser9, which decreases GSK-3β kinase activity and inhibits apoptosis. In another study by Yang et al. (2021), HG decreased the upregulated levels of ROS, MDA, TNF-α and IL-6 in t-BHP-induced Schwann cells, increased the levels of SOD and glutathione (GSH), rebalanced the redox system and increased cell survival by downregulating the NOX2/ROS/TRP/P38 MAPK/NF-κB signalling pathway.

**FIGURE 2:**
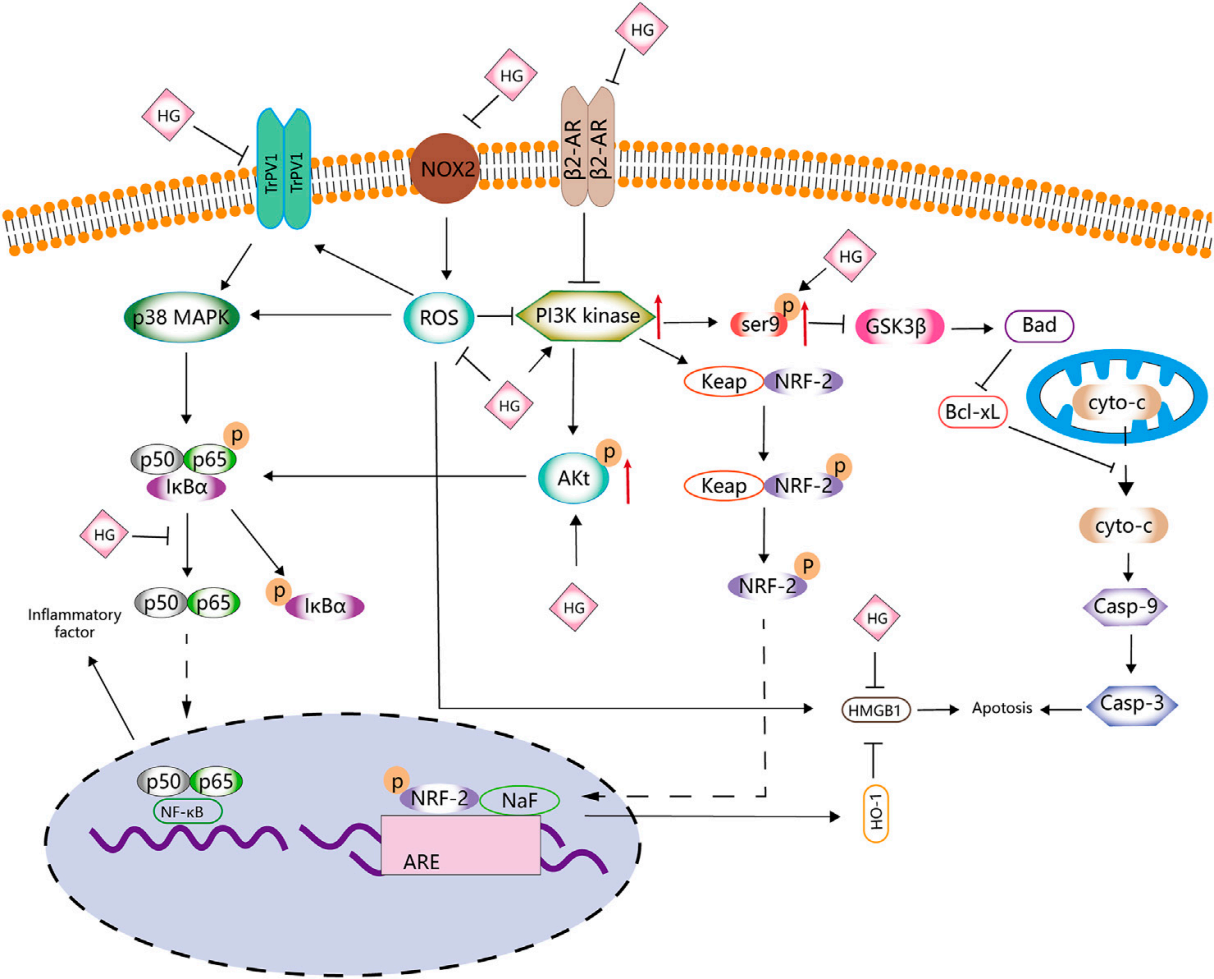
Anti-apoptotic and antioxidative effects of HG. HG inhibits apoptosis and antioxidative stress by affecting the PI3K/AKT, NRF2/HO-1, Ser9p/GSK-3 β, NOX2/ROS, NF-κB and β2-AR signalling pathways.

Therefore, the molecular mechanisms of HG in apoptosis and oxidative stress may involve ROS and PI3k (Figure 2; Table 1). However, to date, studies have only explored some signalling pathways related to ROS and PI3k, and whether HG can be used as a novel PI3k or ROS inhibitor remains unknown.

**TABLE 1:** Anti-inflammatory effects of HG.

Disease	Model	Method	Target genes and proteins	Pathway	Pharmacological target	Reference
Rheumatoid arthritis	DBA/1J mice with collagen-induced arthritis	Intraperitoneal administration: 10 mg/kg/day for 2 weeks	HO-1	PI3K/Akt/Nrf-2 pathway	TNF-α↓, IL-1β↓, MDA↓, GSH↑, caspase-3↓, caspase-9↓, HO-1↑, p-Akt↑ and Nrf-2↑	Duan et al. (2016)
Cerebral ischaemia–reperfusion	Rat models of cerebral ischaemia–reperfusion	Intraperitoneal administration: 10 or 50 mg/kg/day for 4 weeks	TNF-α and ILs	TLR4 pathway	TNF-α↓, IL-1β↓, IL-6↓, IL-18↓, CD14↓, TLR4↓, TAK1↓, NF-κB↓, MIP-2↓ and COX-2↓	Wang Y. et al. (2019)
Depression	Mice with LPS-induced depression and LPS-induced BV2 cells	Cells: 20 μM for 0.5 h; animals: 50 mg/kg/day orally for 3 days	NO and BDNF	BDNF/TrkB pathway, autophagy and endoplasmic reticulum stress	TNF-α↓, IL-1β↓, IL-6↓, NO↓, BIP↓, p-JNK/JNK↓, p-eIF-2α/eIF-2α↓, LC3B-II and BECLIN-1 and BDNF↑	Chen S. et al. (2019)
Rheumatic and autoimmune diseases	Murine peritoneal macrophages	0.01 mM for 18 h	iNOS		iNOS↓	Lee et al. (1999)
Inflammation	RAW264.7 cells	Cells: 1, 10 and 100 μM for 24 h; animals: 10 mg/kg intraperitoneally	iNOS and NF-κB	NF-κB pathway	iNOS↓, NF-κB↓ and NO↓	Kang et al. (1999)
Allergic rhinitis	Mouse model of AR and HNEPCs	Cells: 5, 10 and 20 μM for 24 h; animals: 30, 60 and 120 mg/kg/day orally for 14 days	EGFR, AKT, NO and NF-κB	EGFR/JAK2/c-jun and NF-κB pathways	IgE↓, IL-6↓, IL-8↓, EGFR↓, p-EGFR↓, c-jun↓, p-c-jun↓, iNOS↓, JAK2↓, p-JAK2↓, MUC5AC↓, NF-κB p65↓ and p-NF-κB ↓	Wei et al. (2021)
Intervertebral disc degeneration	Nucleus pulposus cells (NPCs)	Pretreatment: 10, 20 and 40 μM for 2 h	IL-1β, iNOS and NF-κB	NF-κB pathway	NO↓, PGE2↓, iNOS↓, COX-2↓, TNF-α↓, IL-6↓, MMP-3↓, MMP-13↓, ADAMTS-4↓, ADAMTS-5↓, p-NF-κB p65↓ and IκBα↑	Bai et al. (2019)
Neurologic diseases	BV2 cells	0.125, 0.5 and 2.0 μM for 24 h	NF-κB, Nrf2 and HO-1	NF-κB and Nrf2/HO-1 pathways	TNF-α↓, IL-6↓, ROS↓, NO↓, iNOS↓, PGE2↓, COX2↓, HO-1↑, Nrf2↑, NF-κB p65↓ and IκBα↑	Yang S. et al. (2020)
Spinal cord injury	C57BL/6J mice with spinal cord injury	Intraperitoneal administration: 10 mg/kg/day for 6 weeks	iNOS, CD16/32, Agr1, CD206, HMGB1 and HO-1	HO-1 pathway and macrophage transformation	iNOS ↑, CD16/32↑, Agr1↓, CD206↓, HO-1↑, Hmgb1↓, CD4+ T cells↓, CD8+ T cells↓, Ly6G + neutrophils↓ and CD11b + macrophages↓	Zhang et al. (2014)

## Anti-inflammatory effects of higenamine

HG exhibits good anti-inflammatory properties. Duan et al. (2016) found that HG inhibited the activity of TNF-α and IL-1β in a mouse model of collagen-induced arthritis (CIA). Wang X. et al. (2019) reported that HG prevented the overexpression of cytokines (such as TNF-α, IL-1β, IL-6 and IL-18) and inflammation-related factors (such as TLR4, TAK1, NF-κB, MIP-2 and COX-2). These studies strongly validate the anti-inflammatory effects of HG.

Inducible nitric oxide synthase (iNOS) catalyses the production of large amounts of NO, an important cellular signal that is related to many inflammatory responses (Menshikova et al., 2000). Under the action of pro-inflammatory mediators, cells activate the NO pathway and promote the excessive release of NO, further promoting the inflammatory response (Korhonen et al., 2005). In a study, BV2 cells and a mouse model of depression stimulated with LPS released large amounts of NO and corresponding inflammatory mediators, such as TNF-α, IL-1 β and IL-6. However, HG inhibited the release of NO and related inflammatory mediators and the phosphorylation of JNK and EIF2α in the mouse hippocampus and BV2 cells by activating the BDNF/TrkB signalling pathway, thus inhibiting BIP upregulation and reducing NO release (Chen Y. et al., 2019). The levels of p-JNK, p-EIF2α and BIP are closely related to endoplasmic reticulum stress. HG significantly induces the expression of LC3B-II and BECLIN-1, promotes autophagy and reduces the release of NO. However, the autophagy inhibitor 3-MA can block the inhibitory effects of HG on the release of NO and inflammation-related mediators (Chen S. et al., 2019). Therefore, HG may promote autophagy, alleviate endoplasmic reticulum stress and inhibit the release of NO.

Lee et al. (1999) found that HG inhibited the expression of iNOS mRNA in mouse peritoneal macrophages, whereas Kang et al. (1999) revealed that HG exerted these inhibitory effects mainly *via* the NF-κB signalling pathway. iNOS may promote inflammation by activating NF-κB to induce the expression of IL-6 and IL-8 (Seo et al., 2009). NF-κB induces the expression of IL-6 and IL-8 through the transcription factor-binding sites of IL-6 and IL-8 (Zhu et al., 2019). Under normal circumstances, NF-κB binds to the NF-κB inhibitor protein (IκB) to form an inactive complex in the cytoplasm (Wang et al., 2016). When external pro-inflammatory factors stimulate cells, this complex is decomposed, and NF-κB is translocated to the nucleus, resulting in the release of inflammatory mediators (such as IL-1β, TNF-α and IL-6) (Saheb, 1978; Wang et al., 2016; Wang et al., 2018). Bai et al. (2019) found that HG decreased p-p65 expression and IκBα degradation induced by IL-1β in nucleus pulposus cells. The anti-inflammatory effects of HG may be partly attributed to its inhibitory effects on the NF-κB signalling pathway. Yang X. et al. (2020) drew a similar conclusion after treating glial cells with HG. They found that HG significantly inhibited TNF-α, IL-6, ROS, NO (mediated by iNOS) and PGE2 (mediated by COX-2) but promoted NRF-2 and HO-1 in LPS-activated BV2 cells. Therefore, the anti-inflammatory effects of HG may be attributed to its inhibition of NF-κB and activation of the NRF-2/HO-1 signalling pathway.

Wei et al. (2021) found that HG downregulated the expression of IL-6 and IL-8 via the iNOS, NF-κB and c-jun pathways. HG promoted the expression of AKT1 and p-AKT1 in histamine-treated human nasal epithelial cells (HNEpCs) and inhibited the expression of epithelial growth factor receptor (EGFR), p-EGFR, c-jun, p-c-jun, iNOS, JAK2 and p-JAK2. These findings suggest that HG inhibits the EGFR/JAK2/c-jun signalling pathway by activating AKT1, thereby inhibiting the expression of iNOS, IL-6 and IL-8 and the release of NO.

Macrophages are an indispensable type of immune cell involved in the inflammatory response. They have high plasticity and can rapidly adjust to changes in the microenvironment. They have two phenotypes with different functions, namely, M1 and M2 macrophages (Chawla et al., 2011). M1 macrophages increase the expression of pro-inflammatory factors and ROS production, whereas M2 macrophages are anti-inflammatory and possess wound-healing properties (Oishi and Manabe, 2018). Zhang et al. (2014) found that macrophages in the spine of mice with spinal injury polarised from the M1 to the M2 phenotype in a time-dependent manner after intragastric administration of HG. The expression of related pro-inflammatory cytokines, such as IFN-γ and TNF-α, was significantly lower and that of anti-inflammatory cytokines, such as IL-4 and IL-10, was significantly higher in HG-treated macrophages than in control macrophages. In addition, the proportion of immune cells, such as CD4+ T cells, CD8+ T cells, Ly6G + neutrophils and CD11b + macrophages, was lower, and HG promoted the expression of HO-1 and inhibited the expression of HMGB1.

**FIGURE 3:**
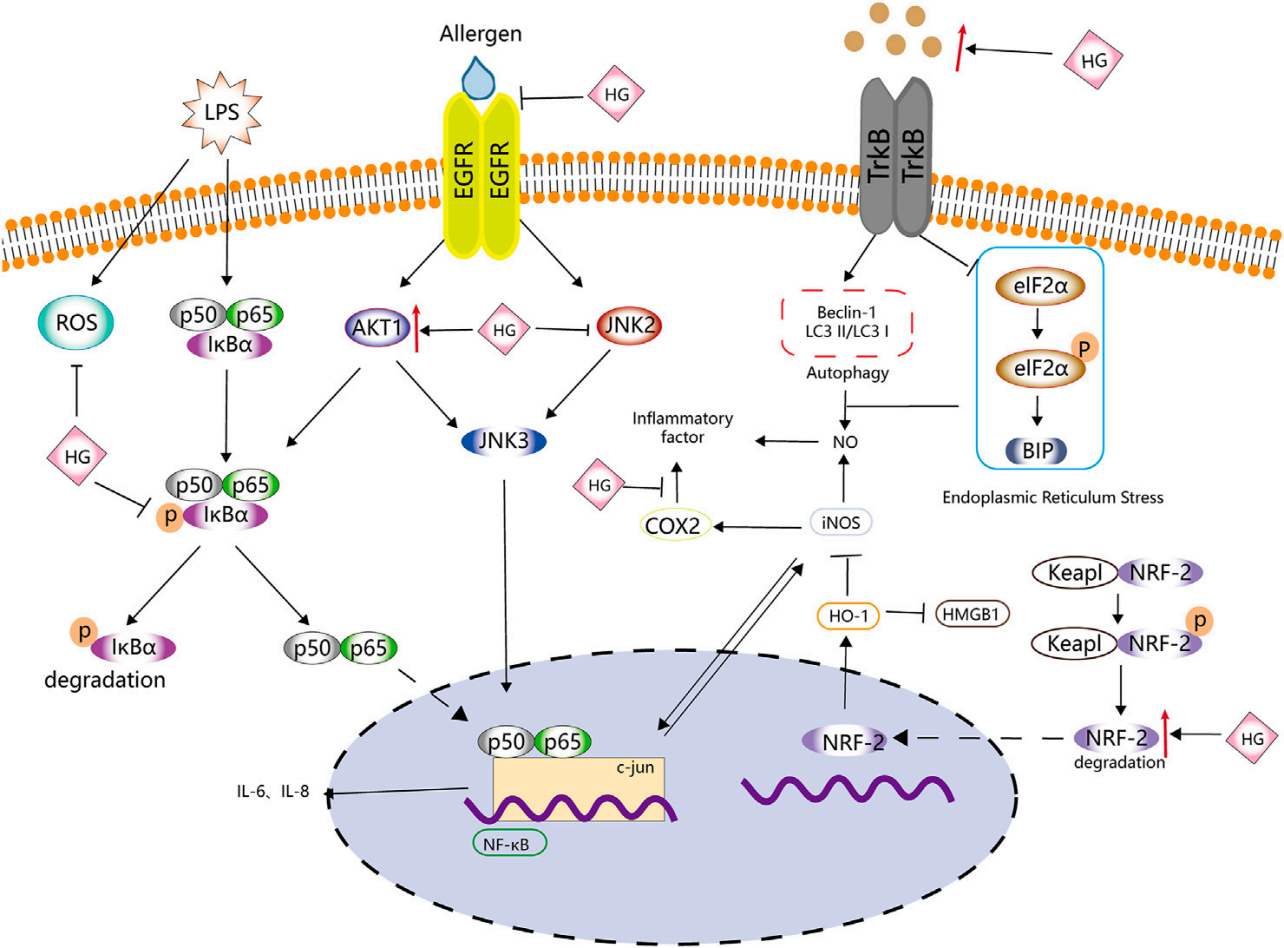
Anti-inflammatory effects of HG. HG inhibits inflammation by affecting ROS, NF-κB, iNOS and NRF-2-related inflammatory signalling pathways and the related upstream and downstream targets.

A schematic diagram demonstrating the anti-inflammatory activity of HG is shown in Figure 3, Table 2 . HG can effectively inhibit inflammation and affect multiple inflammation-related signalling pathways. It also acts on immune cells, such as macrophages and T cells, and can be used as a natural anti-inflammatory drug with fewer side effects to treat inflammation-related diseases. However, there is a lack of relevant clinical trials to confirm the clinical anti-inflammatory effects of HG.

**TABLE 2:** Anti-apoptotic and antioxidant effects of HG.

Disease	Model	Method	Target genes and proteins	Pathway	Pharmacological target	Reference
Ischaemia–reperfusion (I/R) injury	Rat models of myocardial ischaemia and reperfusion injury	Intraperitoneal pretreatment: 1, 5 and 10 mg/kg for 1and 24 h	HO-1	HO-1 pathway	Cytochrome C↓, caspase-3↓, Bax↓, Bcl-2↑ and HO-1↑	Lee et al. (2006)
Ischaemic injuries	C6 cells and rats with ischaemic injury	Cells: 50 μM for 12 h; animals: intraperitoneal pretreatment with 10 mg/kg 24 h	HMGB1, HO-1, PI3K and AKT	PI3K/Akt/NRF-2 pathway	HO-1↑, p-Akt/t-Akt↑, NFR-2 (cytosolic)↓, NFR-2 (nucleus)↑, HMGB1↓, Bax↓, Bcl-2↑ and caspase-3↓	Ha et al. (2012)
Intestinal ischemia and reperfusion syndrome	Intestinal epithelial (IEC-6) cells of mice and mice with ischaemic injury	Cells: 0–150 μM for 0–24 h; animals: 10 mg/kg intraperitoneally	NRF-2, HO-1 and HMGB1	NRF-2/HO-1/HMGB1 pathway	HO-1↑, TNF-α↓, IL-6↓, MPO↓, HMGB1↓, F4/80 + cells↓ and Ly6G+ cells↓	Liu et al. (2015)
Ischaemic cardiac injury	C57BL/6J mice with myocardial ischaemia and reperfusion injury and neonatal rat ventricular myocytes (NRVMs)	Cells: 100 μM; 24 h; animals: intraperitoneal pretreatment with 10 mg/kg for 24 h	β2-AR, PI3K and AKT	β2-AR/PI3K/AKT pathway	Cleaved caspase 3↓, cleaved caspase 9↓ and p-Akt/Akt↑	Wu et al. (2016)
Diabetic gastroparesis	Rat models of DGP and gastric smooth muscle cells (SMCs)	Intraperitoneal administration: 10 mg/kg/day for 3 weeks	β2-AR, PI3K and AKT	β2-AR/PI3K/AKT pathway	p-21↓, p-GSK3β↓, p-BAD↓, caspase-3↓, caspase-9↓, p-Akt/Akt↑, p-PI3K/PI3K↑, KI67↑ and PCNA↑	An et al. (2017)
Intervertebral disc degeneration	Human nucleus pulposus cells (HNPCs)	Pretreatment: 10, 20 and 40 μM for 2 h	ROS, PI3K and AKT	ROS/PI3K/AKT pathway	Cleaved caspase-3↓, Bax↓, Bcl-2↑, ROS↓ and p-Akt/Akt↑	Zhu X. et al. (2021)
Stroke	Neuronal cells of rats	Pretreatment: 30 and 60 μM for 24 h	ROS, PI3K, AKT, NRF-2 and HO-1	PI3K/Akt/Nrf2/HO-1 pathway	ROS↓, MDA↓, SOD↑, GPx↑, caspase-3↓, Bax↓, Bcl-2 and p-Akt/Akt↑, HO-1↑ and NFR-2↑	Zhang Y. et al. (2019)
Alzheimer’s disease	Albino Wister rats	Oral administration: 25, 50, 75 and 100 mg/kg/day for 42 days	AChE, Akt, GSK-3β and ROS	Akt/GSK-3β pathway	Acetylcholinesterase activity↑, APP↓, Aβ1-42↓, β-secretases↓, γ-secretases↓, Bax↓, Bad↓, caspase-3↓, caspase-9↓, cytochrome C (mitochondrial fraction)↑, Bcl-2↑, Bcl-xL↑, cytochrome C (cytosolic fraction)↓, ROS↓, MDA↓, SOD↑, GPx↑, p-Akt↓, Ser9 and pGSK-3β↑ and GSK-3β kinase activity↓	Yang X. et al. (2020)
Neuropathic pain	Rat Schwann cells (RSC96) and rat models of chronic constriction injury (CCI)	Cells: Pretreatment with 100, 200 and 400 μM for 24 h; animals: 25, 50 and 100 mg/kg/day orally for 3 weeks	ROS, TRPV1 and NOX2	NOX2/ROS/TRP/P38 MAPK/NF-ĸB pathway	ROS↓, MDA↓, SOD↑, GSH↑, TNF-α↓, IL-6↓, Bcl-2/Bax↑, cyt-c↑, cleaved caspase 3/caspase 3↓, Nox2↓, TRPA1↓, TRPV1↓, p-p38 MAPK↓ and p-NF-ĸB↓	Yang et al. (2021)
Cancer	C6 glioma cells	0–200 μM for 24, 48 and 72 h	NF-κB	NF-κB pathway, phosphoinositide-3-kinase/protein kinase B pathway and caspase cascade	Nuclear translocation of NF-ĸB↓, B-cell lymphoma 2↓, BCL2- associated X protein↑ and cysteine–aspartic proteases-3 and -9↑	Geng and Ou, (2021)
MLL-rearranged leukaemia	MLL-rearranged leukaemia cells	0–100 μM for 72 h	LDS1, HoxA9 and Meis1	LDS1-related pathway	H3K4me1↑, H3K4me2↑, H3K4me3 (−) and LDS1↓, p53↑, HoxA9↓ and Meis1↓	Fang et al. (2021)

## Anti-fibrotic effects of higenamine

Oral administration of HG can increase the levels of plasma fibrinogen and fibrinogen/fibrin degradation product (FDP) and the prothrombin time (PT) (Yun-Choi et al., 2002), indicating that HG possesses anti-fibrotic properties. Transforming growth factor-β1 (TGF-β1) is important for fibrosis in many tissues and organs (Kim et al., 2018). Deng et al. (2020) found that the pathogenesis of cardiorenal syndrome in rats involves ventricular remodelling and renal fibrosis, and HG can systematically improve cardiorenal function. In addition, HG inhibits collagen synthesis in neonatal rat cardiac fibroblasts and cardiomyocyte hypertrophy induced by TGF-β1/NF-kB by downregulating the phosphorylation of ASK1/MAPK (ERK, P38)/NF-κB, thus inhibiting cardiac and renal fibrosis. Furthermore, HG can block Smad2/3 phosphorylation and nuclear translocation stimulated by TGF-β1, indicating that HG inhibits the TGF-β/Smad signalling pathway, which is associated with the activation of cardiac fibroblasts and fibrosis (Zhu J. X. et al., 2021). However, the specific mechanism of HG underlying its inhibitory effects on cell fibrosis remains unclear and warrants further investigation.

## Anti-platelet activity of higenamine

Although platelets play an important role in haemostasis, endogenous stimuli can easily activate platelets, resulting in their aggregation and thrombus formation. Yun-Choi et al. (2001) induced acute thrombosis in mice by injecting a mixture of collagen and epinephrine and found that HG inhibited platelet aggregation. They speculated that HG may compete with epinephrine at the α2-AR site on platelets because it is a structural analogue of catecholamines. However, the inhibitory effects of HG on epinephrine-induced thrombosis are much lower than that of α2-AR inhibitors, such as phentolamine and yohimbine (Pyo et al., 2007).

**FIGURE 4:**
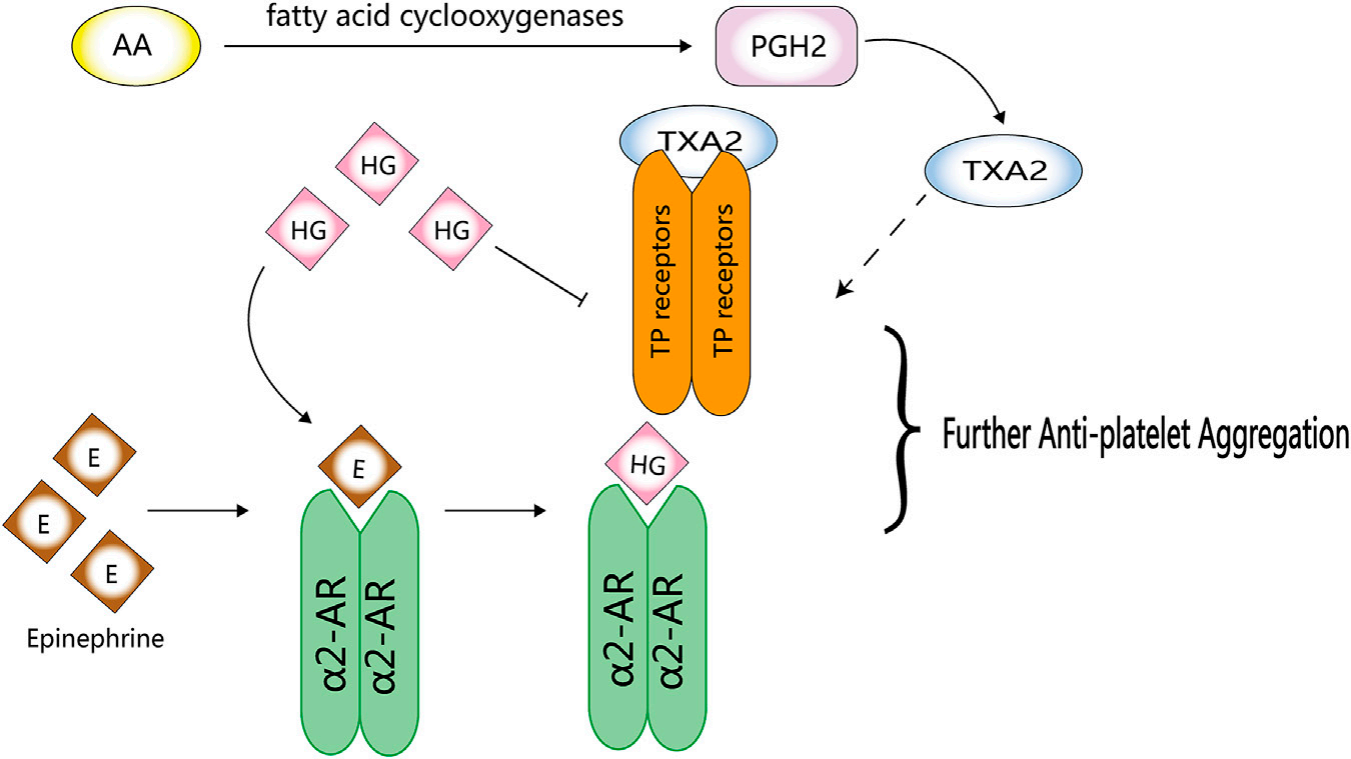
Anti-platelet activity of HG. HG can competitively inhibit the binding of epinephrine to α2-AR and directly act on TP receptors to inhibit platelet aggregation.

HG can inhibit platelet aggregation by directly acting on TP receptors; however, its inhibitory effects on the synthesis of thromboxane A2 (TXA2) from arachidonic acid (AA) remain clear (Pyo et al., 2007) (Halushka, 2000). The analogues of HG, YS-49 and YS-51, not only directly act on TP but also significantly inhibit the synthesis of TXA2 from AA (Pyo et al., 2007) (Figure 4). Compared with the natural HG, (R)-(+)-HG and (S)-(-)-HG, two synthetic stereoisomers of HG, have enhanced inhibitory effects on platelet aggregation (Pyo et al., 2008).

In some thrombus-induced diseases, the use of anticoagulants can further prolong the PT, activated partial thrombin time (aPTT) or thrombin clotting time (TCT) (Onaya et al., 1998). An increase in PT and aPTT is closely related to the possibility of bleeding complications (Petitti et al., 1989). Yun-Choi et al. (2002) found that LPS induced disseminated intravascular coagulation (DIC) in mice, resulting in the prolongation of PT, aPTT and TCT. However, oral administration of HG prevented this increase, indicating that HG is less likely to cause bleeding complications than anticoagulants.

## Adrenergic receptor ligands

### α1-Adrenergic receptor

HG can reduce blood pressure in both normotensive and hypertensive animal models. Praman et al. (2012a) reported that HG increased the heart rate but not blood pressure in rats, which can be associated with the ability of HG to reduce peripheral vascular pressure. Moreover, the antagonistic effects of α1-AR may also be a factor. Phenylephrine induces HEK293A cells to activate α1-AR, which in turn promotes the activation and dissociation of βγ and α subunits of the Gαq protein, leading to the intracellular release of calcium from the sarcoplasmic reticulum. As a result, IP3 and calcium are accumulated intracellularly, and the ERK1/2 signalling pathway is activated. This process can be reversed by HG. Zhang N. et al. (2019) reported that HG decreases the contractile response of the mesenteric artery. HG can prevent the elevation of blood pressure by antagonising α1-AR, thereby regulating the ERK1/2 signalling pathway, and inhibiting the accumulation of intracellular IP3 and calcium.

### α2-Adrenergic receptor

HG can inhibit vasoconstriction of the tail vein and promote the rate of blood flow in mice at low temperatures (Guan et al., 2019). The AMPK/eNOS/NO signalling pathway plays an important role in regulating energy metabolism. Endothelial cells produce NO, a vasodilator that modulates vascular tension. *In vitro* treatment of human dermal microvascular endothelial cells with HG can suppress the AMPK/eNOS/NO signalling pathway and NO production (Guan et al., 2019). In addition, HG can inhibit vasoconstriction and AMPK/eNOS/NO signalling. Low temperatures can lead to a rapid increase in ROS in vascular smooth muscle cells (VSMCs) of the skin, activate the Rho/Rho-kinase signalling pathway and promote the migration of intracellular static α2C-AR to the cell surface, resulting in the contraction of vascular smooth muscle (Bailey et al., 2004; Honda et al., 2007; Jeyaraj et al., 2012). Furthermore, the phosphorylation of protein tyrosine kinase (PTK) and its binding to actin also mediate vasoconstriction induced by hypothermia (Furspan et al., 2005). HG can inhibit hypothermia-induced activation of the ROS/α2C-AR and PTK9 signalling pathways, which may be the mechanism underlying the inhibition of vascular smooth muscle contraction (Guan et al., 2019). In addition, HG can inhibit adrenaline-induced platelet aggregation in humans and rats by blocking α2-AR (Yun-Choi et al., 2001).

### β1-Adrenergic receptor

HG is considered a β1-AR agonist. Kimura et al. (1994); Kimura et al. (1996) reported that HG increased cardiac contractility, and its positive chronotropic effects were antagonised by propranolol (a non-selective β1- and β2-adrenergic antagonist) and practolol (a selective β1-adrenergic antagonist) but not by butoxamine (a selective β2 receptor antagonist). Therefore, HG may activate β1-AR in the atrium and mediate cardiac inotropic and chronotropic effects, which may be attributed to the responses mediated by β1-AR. Similar to isoproterenol, HG controls the electrophysiology of the heart by affecting sinoatrial node cells and does not induce arrhythmia (Wang Y. et al., 2019). Praman et al. (2012b) reported that HG activates β1-AR instead of β2-AR, resulting in a positive chronotropic effect.

### β2-Adrenergic receptor

HG can act on β2-AR, which belongs to the G protein-coupled receptor (GPCR) family and is mainly found on smooth and skeletal muscle cells and in the liver. β2-AR regulates blood flow, energy storage, bronchial smooth muscle relaxation, liver glucose production and muscle glucose uptake (Kato et al., 2017). HG can be used as a novel β2-AR agonist because of its structural similarity to catecholamines (Hudzik et al., 2021). Wu et al. (2016) reported that β2-AR can serve as a molecular target for the protective effects of HG on cardiomyocytes. In addition, Kato et al. (2017) found that HG can promote glucose uptake in immortalised rat skeletal muscle cells (L6 cells). These effects can be attributed to the action of HG on β2-AR, suggesting that HG is a potential drug candidate for the management of various health conditions, such as diabetes and obesity. Cell proliferation and differentiation are strongly associated with the ERK1/2 signalling pathway (Nishimoto and Nishida, 2006). Zhang et al. (2021) reported that HG activates the ERK1/2 signalling pathway. They found that phosphorylation of ERK1/2 in cardiomyocytes was time-dependent, which peaked after 5 min of stimulation in the treatment group but after 60 min in the control group. In addition, HG-induced phosphorylation of ERK1/2 was not sensitive to GI protein inhibitors, indicating that the effects of HG may not be realised by G proteins. On the contrary, HG may induce ERK1/2 phosphorylation via EGFR transactivation and rely on β-arrestin1/2, which aids in understanding the mechanism of action of HG on β2-AR (Zhang et al., 2021).

HG can act as a vasodilator and smooth muscle relaxant and increase perfusion. Park et al. (1984) reported that HG increased the rate of relaxation and shortened the time to peak and the duration of contraction in a dose-dependent manner. However, these effects were competitively blocked by propranolol (non-selective β1- and β2-adrenergic receptor blockers). Therefore, HG may serve as a β-AR agonist. Furthermore, Bai et al. (2008) validated the β2-AR agonist activity of HG at molecular and cellular levels for the first time. They found that HG acted as a tracheal muscle relaxant, which reduced the severity of bronchial stenosis both *in vivo* and *in vitro*. Tsukiyama et al. (2009) and Ueki et al. (2011) isolated HG from the fruit of *Nandina domestica* Thunberg and found that HG stimulated β2-AR and had relaxing effects on the corpus cavernosum of Sprague-Dawley (SD) rats and bronchial smooth muscle. Kam et al. (2012) demonstrated that HG caused the rat corpus cavernosum to relax in a dose-dependent manner and enhanced the relaxation response of the corpus cavernosum induced by a PDE-5 inhibitor (a drug for the treatment of penile erectile dysfunction). This vascular smooth muscle relaxation response is mediated by cGMP and cAMP. In rats, the increased expression of cGMP and cAMP in cells causes dephosphorylation of the myosin light chain, resulting in relaxation of the corpus cavernosum smooth muscle. (Ballard et al., 1998) Kam et al. (2012) found that the expression of cGMP and cAMP increased under the action of HG, indicating that the mechanism of action of HG may be associated with the β-AR/cAMP and guanylate cyclase/cGMP signalling pathways.

**FIGURE 5:**
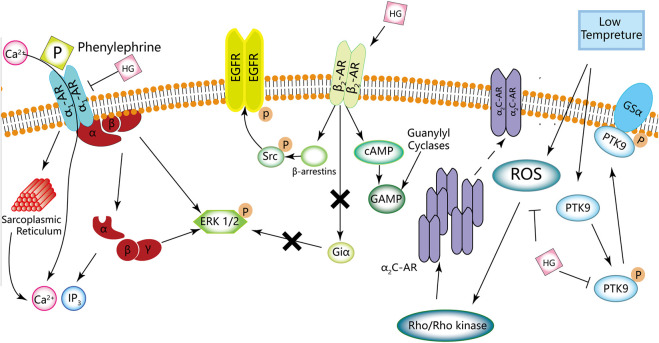
Adrenergic effects of HG. HG may act as a β-AR agonist and an α-AR inhibitor.

A schematic diagram demonstrating the effects of HG on adrenergic receptors is shown in Figure 5, Table 3 . HG acts as an agonist of β1-/β2-AR. Because HG is structurally similar to catecholamines, it can activate both β1-AR and β2-AR; however, the exact mechanism of action of HG remains unknown (Chen Y. et al., 2019).

**TABLE 3:** Pharmacological effects of HG on adrenoceptors.

Disease	Model	Method	Target genes and proteins	Pathway	Pharmacological target	Reference
Hypertension	HEK293 cells, *ex-vivo* rat mesenteric artery and rats with spontaneous hypertension	Cells: 0.1, 1, 10 and 100 μM for 5, 15 and 30 min; *ex-vivo*: 10 μM; animals: 1–3 μg/kg intravenously	α1-AR	ERK1/2 pathway	Ca2+↓, IP3↓ and p-ERK/ERK↓	Zhang N. et al. (2019)
Raynaud’s phenomenon	HDMECs and rat models of cold-induced cutaneous vasoconstriction	Cells: 20 μM; 2 h; animals: 18, 36 and 72 μg/kg intravenously	ROS, PTK9, NO and α2C-AR	ROS/α2C-AR and PTK9 pathways	NO↓, eNOS↓, p-eNOS↓, Akt1↓, p-Akt1↓, AMPK α1↓, p-AMPK α1↓, ROS↓, α2C-AR (intracellular)/α2C-AR (membrane) ratio↑ and PKT↓	Guan et al. (2019)
Bradycardia	Ventricular myocytes	0–10 μM; 0–5 min	β1-AR	—	ICa-L↑, Iks↑ and heart rate ↑	Wang X. et al. (2019)
—	Neonatal rat cardiac fibroblasts (NRCFs) and HEK293A cells	0–10 μM, 0–30 min	β2-AR, β-arrestin1/2 and EGFR	β2-AR/β-arrestin/EGFR/ERK pathway	p-ERK1/2/ERK1/2↑, p-EGFR↑, p-Scr↑, β-arrestin↑ and Cleaved-caspase 3↓	Zhang et al. (2021)
Erectile dysfunction	Corpus cavernosum of male Sprague-Dawley rats	0.1, 1 and 10 μM	β2-AR, cGMP and cAMP	β-adrenoceptor/cAMP and guanylate cyclase/cGMP pathways	cGMP↑ and cAMP↑	Kam et al. (2012)

## Other functions

### Regulation of cellular electrophysiology

HG can affect the movement of ions inside and outside the cell. In the distal colonic mucosa of guinea pigs, HG can produce alterations in the short circuit current and transepithelial conductance and inhibit electrogenic Na^+^ absorption while stimulating electrogenic K^+^ and Cl^−^ secretion via β2-AR stimulation (Liu et al., 2000). Wang X. et al. (2019) examined the electrophysiological effects of HG on ventricular and sinoatrial node cells and found that HG increased the L-type Ca^2+^ current (I_Ca-L_) and slowly activating delayed rectifier potassium current (I_Ks_) in a concentration- and voltage-dependent manner and enhanced the synchronisation of I_Ca-L_ and I_Ks_. However, HG does not affect the sodium channel (I_Na_) and fast delayed rectifier potassium channel (I_Kr_). Sinoatrial node cells have a higher sensitivity to HG than ventricular cells. In PC12 cells (rat adrenal pheochromocytoma cells), HG can suppress the levels of dopamine and decrease the activity of tyrosine hydroxylase (TH). HG is related to α-AR antagonists, which possess calcium channel-blocking activity. In a study by Shin et al. (1999), HG reduced the concentration of intracellular calcium for 12–24 h, which returned to normal at 48 h. In addition, the kinetic model of Ca^2+^ was similar to that of TH. Therefore, HG may affect the transport of potassium, sodium, calcium and chloride ions; however, the specific underlying mechanisms warrant further investigation.

### Lipid-lowering effects

Dietary supplements containing HG can enhance fat decomposition and energy consumption (Lee et al., 2013). HG contributes little to the increase in these haemodynamic variables but promotes the consumption of free fatty acids in plasma and energy, whereas supplements can moderately increase the heart rate (approximately by 3 bpm) and systolic blood pressure (approximately by 12 mmHg).

## Combination therapy

### Combination of higenamine with cucurbitacin B

The combination of *Trichosanthes kirilowii* Maxim and Fuzi has been used in ancient medicinal formulas (Jin et al., 2018). *Trichosanthes kirilowii* Maxim contains Cu B, an anticancer tetracyclic triterpenoid (Garg et al., 2017), whereas Fuzi contains HG. When used in combination, HG and Cu B have synergistic anticancer effects (Geng and Ou, 2021). Jin et al. (2018) used network pharmacology to predict the possible mechanisms by which HG enhances the antitumour activity of Cu B. KEGG pathway analysis revealed that AKT may be the primary target of Cu B. In addition, compared with Cu B monotherapy, combination therapy with HG and Cu B significantly increased the number of stagnant cells in the G2/M phase. Based on a study by Maddika et al. (2008), this enhanced antitumour activity can be attributed to the inhibition of CDK2, as evidenced by an increase in Bax expression and a decrease in Bcl-2 expression. In addition, Jin et al. (2018) reported that the combined effects of Cu B and HG on the proliferation of breast cancer cells resulted in cell cycle arrest and induced apoptosis, which may be attributed to the activity of CDK2.

### Combination of higenamine with 6-gingerol

In traditional medicine, a combination of Fuzi and ginger is commonly used in the management of heart failure and coronary artery disease (Wen et al., 2020b). 6-GR, a major bioactive constituent of ginger, exerts anticancer, anti-inflammatory and antioxidant effects through various biological pathways involved in apoptosis, cell cycle regulation, cytotoxicity and angiogenesis inhibition (Wang et al., 2014). HG/6-GR may exert therapeutic effects by improving mitochondrial dysfunction (Wen et al., 2020a). Chen et al. (2013) reported that combination therapy with HG and 6-GR significantly attenuated doxorubicin-induced oxidative stress and apoptosis in cardiomyocytes, increased Bax expression and decreased cytochrome C release and caspase-3 activity. However, these effects were inhibited by LY294002 (a PI3K/AKT inhibitor) (Chen et al., 2013). Therefore, the PI3K/AKT signalling pathway may be involved in the therapeutic effects of HG/6-GR on cardiomyocytes. Cell proliferation and apoptosis are regulated by the PGC-1α, SIRT3 and PI3K/AKT signalling pathways (Tsang et al., 2003; Jing et al., 2011; Li et al., 2022). PGC-1α and PPARα (co-activator of PGC-1α) are critical regulators of energy metabolism (Yang et al., 2015). PGC-1α regulates the transcription of SIRT3, which affects the expression of ATP in the mitochondria (Jing et al., 2011). Combination therapy with HG and 6-GR can alleviate doxorubicin-induced mitochondrial energy metabolism disorder and respiratory function impairment in H9c2 cells. Upregulation of the PPARα/PGC-1α/SIRT3 signalling pathway, which promotes mitochondrial energy metabolism, may exert protective effects against heart failure. In addition to the PPARα/PGC-1α/SIRT3 signalling pathway, HG/6-GR can upregulate the LKB1/AMPK/SIRT1 axis and inhibit mitochondrial dysfunction (Wen et al., 2020b).

## Conclusion

HG possesses anti-platelet, anti-inflammatory, antioxidant, anti-apoptotic, anti-fibrotic and lipid-lowering activities. To date, most studies have focused only on the effects of HG on the classical pathways and target genes of diseases, and the exact site of action of HG remains unknown. HG is a ligand of adrenergic receptors that regulates the relaxation of vascular smooth muscle, tracheal smooth muscle, myocardium and corpus cavernosum and can be used to treat coronary heart disease, asthma and erectile dysfunction. However, conflicting findings have been reported regarding the effects of HG on adrenergic receptors. In general, HG can be used as both a β-AR agonist and an α-AR antagonist. However, regarding adrenergic receptor subtypes, some researchers speculate that HG acts on β1-AR, whereas others speculate that HG acts on β2-AR, which warrants further investigation. To date, most studies have employed cell and animal models, which lack the support of clinical trials. Although HG is a natural compound, only a few studies have investigated its safety. At present, there are three routes of administration for HG: intravenous administration, intraperitoneal injection and oral administration. Animals can tolerate intraperitoneal administration of up to 50 mg/kg/day. A maximum dose of 120 mg/kg/day has been orally administered in a small number of studies. Intravenous injection may be an effective route of administration because HG has a low oral utilisation rate. However, only a few studies have used intravenous administration, with the highest dose of 72 μg/kg. At present, the maximum tolerable dose for intravenous injection of HG in humans is 24 μg/kg. However, a few studies with small sample size have investigated the safe dosage of HG in clinical practice. Therefore, further studies with large sample size should be conducted to determine the safety of HG in humans and provide a reference dosage for clinical research, which will eventually contribute to the design of new, safer, more effective drugs based on HG.
